# Gender differences in care-seeking behavior and healthcare consumption immediately after whiplash trauma

**DOI:** 10.1371/journal.pone.0176328

**Published:** 2017-04-25

**Authors:** Artur Tenenbaum, Lena Nordeman, Katharina S. Sunnerhagen, Ronny Gunnarsson

**Affiliations:** 1 Hälsan & Arbetslivet, Occupational Health Care Unit, Region Västra Götaland, Gothenburg, Sweden; 2 Department of Public Health and Community Medicine Institute of Medicine, The Sahlgrenska Academy, University of Gothenburg, Gothenburg, Sweden; 3 Research and Development Center Södra Älvsborg Närhälsan, Primary Health Care, Region Västra Götaland, Gothenburg, Sweden; 4 Institute of Neuroscience and Physiology, Department of Health and Rehabilitation, Unit of Physiotherapy, University of Gothenburg, Gothenburg, Sweden; 5 Institute of Clinical Neuroscience—Rehabilitation Medicine, University of Gothenburg, Gothenburg, Sweden; 6 Cairns Clinical School, College of Medicine and Dentistry, James Cook University, Cairns, Australia; Johns Hopkins University Bloomberg School of Public Health, UNITED STATES

## Abstract

**Objective:**

The aim was to study gender differences in care-seeking behavior and treatment provided immediately after whiplash trauma.

**Methods:**

Participants were residents from a defined geographical area, Skaraborg County in the southwestern part of Sweden. A cohort of 3,368 persons exposed to whiplash trauma and attending a healthcare facility immediately after the trauma between 1999 and 2008 were identified in a database. Information about gender, age, time elapsed prior to seeking care, type of healthcare contact, initial treatment provided and eventual hospitalization time was retrieved.

**Results:**

Women sought care later than men (p = 0.00074). Women consulted primary healthcare first more often than men, who more often first sought hospital care (p = 0.0060). There were no gender differences regarding the type of treatment after trauma. Women had longer hospital admission than men (p = 0.022), indicating their injuries were at least similar to or worse than men’s.

**Conclusion:**

Women sought healthcare later than men after whiplash trauma. Although not directly investigated in this study, it raises the question if this may reduce their probability of getting financial compensation compared to men.

## Introduction

The incidence of whiplash associated disorders (WAD) varies and its annual incidence is estimated to be 100–320 per 100 000 residents in Sweden [1,2], 80–420 per 100 000 inhabitants in Denmark, Spain and France [3–6] and 400 per 100 000 residents in the US [7]. The annual costs are estimated to be 10 billion euros in Europe [8] and 420 million euros in Sweden, the highest cost being loss of production [4]. The clinical presentation varies but often consists of neck pain, headache and vertigo, as well as cognitive disturbances (memory and attention), and stress intolerance [9–13]. In the longer perspective 30–50% of patients exposed to a whiplash trauma face chronic health problems [2,4, 14–20]. About 2.5% of the traffic accidents leading to medical disability and insurance claims are from persons injured in accidents in modern cars that have whiplash protection [4], indicating that the problem of whiplash injuries is not going to disappear in the near future.

### Assessment of signs and symptoms after whiplash trauma

The timing of seeking healthcare following an injury is one factor that affects the outcome of an injury assessment made by insurance companies. Insurance companies and courts often refer to the 72—hour rule, stating that seeking healthcare more than 72 hours after trauma means that symptoms are not related to the whiplash trauma. The Swedish Whiplash Commission, with assistance of an expert group from the Swedish Medical Society, noted that although it is reasonable to assume that symptoms related to a whiplash trauma should be apparent within a few days after trauma, there is no scientific evidence to support a definite time limit for seeking healthcare [4]. Another factor influencing the outcome of injury assessment after whiplash trauma is the quality of documentation available from medical charts [4]. Early clinical investigation and proper documentation are essential to settle insurance claims and focus on the management of symptoms according to a Swedish structured management program for the treatment of symptoms after a whiplash trauma [15, 17, 21].

### Gender differences in healthcare utilization

In a cross-sectional study by Osiaka et.al [22] of 1.6 million inhabitants in the region of Västra Götaland, a well-defined geographical region in south-western Sweden noted that women were more likely to receive more accessible, less expensive primary healthcare, while men were more likely to receive more expensive specialist inpatient care. Another study by Raine [23] suggest that women generally receive more low cost primary healthcare resources than men. Consequently it has been shown that men sometimes receive more resource and treatment than women, even when their illness/injury is the same [24]. Women referred to a cardiac rehabilitation program are less likely to be instructed on secondary prevention strategies [25–27]. Results from the Framingham Heart Study support the existence of gender-differences in rehabilitation after stroke, with poorer outcome observed in women [28].

### Gender differences in whiplash injuries

Holm et al [12] showed there has been an increase in visits to emergency rooms due to whiplash trauma in the western world over the past 30 years, with women seeking care for WAD more often than men. They also found that headrests/car seats constructed to limit head retraction during rear-end collisions have been shown to be beneficial, especially to women [12]. Furthermore, women were found to have more symptoms related to whiplash trauma compared to men [29].

Gender differences in care-seeking behavior and overall management of patients after whiplash trauma can have a large impact on rehabilitation and financial compensation in the event of chronic health problems. It has previously been shown that gender differences concerning care-seeking behavior after whiplash trauma exists if the trauma is work related [30]. However, gender differences in care-seeking behavior for non-work related WAD is not yet studied. Hence, further elucidation of gender differences in patients exposed to a whiplash trauma is warranted.

This study aims to investigate gender differences in care-seeking behavior regarding time elapsed before seeking healthcare, type of healthcare facility sought, treatment given and length of hospital stay immediately after whiplash trauma.

## Methods

Injury registration in Skaraborg County, a part of the larger region of Västra Götaland in south-west Sweden began in 1997, encompassing four hospitals, four emergency primary healthcare units and 25 ordinary primary healthcare facilities. The participating healthcare facilities represent all healthcare facilities in this geographical area treating patients immediately after a whiplash trauma. Skaraborg County is a rural area with four mid-sized cities.

The process of registering data to the database was as follows: after the injured patient gave consent, information provided by the patient or attending person about the trauma was entered into the database. The physician in charge documented the diagnosis according to ICD-10 and recorded treatment, including hospitalization.

A patient’s first attendance at any healthcare facility related to the trauma was registered irrespective of delay after exposure to the trauma. To minimize dropouts, a first check was made by the secretary typing out the medical record and a second check was performed by comparing the administrative file of all patients and the cashier’s book. Missing cases were checked by random selection and detailed check of events during 36 days per year. All visits to the clinics due to any type of injury were compared with register entries, showing that 80% of all presenting injuries were properly included into the database during the ten-year period. Of these, 15% were due to traffic collisions.

All hospitals and healthcare facility dealing with acute injuries coded and classified patients according to three systems–the Nordic Medicinalstatistisk Committee's (NOMESCO) classification, European Home and Leisure Accident Surveillance System (EHLASS), and ICD-10. Registration was carried out using computer software developed by the Swedish National Board of Health and Welfare. Quality control of the process was performed on a regular basis. Creation and maintenance of the database was funded by Region Västra Götaland and The Swedish National Board of Health and Welfare. One of the aims of constructing the database was to use it as a tool for research.

The present study extracted the following data: gender, age, type and circumstances of the collision, time elapsed before seeking care, healthcare contact, treatment, and days of hospitalization from the database for the period 1999–2008. Changes in the population in Skaraborg were small over this 10-year period. There were no large changes in health policy or practices between 1999 and 2008 that may have influenced the results. The Regional Ethical Review Board of Gothenburg, Sweden approved the study (Registration number/Dnr: 138–08 Decision date 2008-04-28).

### Statistical analysis

A chi-square test was used to analyze differences between men and women in care-seeking behavior and the type of care received. Mann Whitney’s U-test was used to analyze differences between men and women concerning elapsed time between trauma and seeking care. Finally, a student’s t-test was used to compare the length of hospital stay between genders.

Multivariate linear regression was used to analyze the relation between the dependent variable patient delay between trauma and seeking care (days) and gender while adjusting for confiding variables such as age, work related accident and car traffic accident. Prior the multivariate linear regression, the variables were evaluated for the assumptions of linear regression. The dependent variable vas not normally distributed and subsequently transformed to ranked normal score using Blom´s formula [31]. Spearman Rank Correlation was used to decide which variables to put into the multivariate regression. All variables with significant correlation p <0.05 with the dependent variable in Spearman’s rank correlation was put forward into the final multivariate linear regression. The exact value of the beta coefficient as such makes no sense since the dependent variable is transformed. Hence, it is presented as being above or below zero to indicate the direction of a correlation. Statistical significance was set at p< 0.05. The statistical software IBM SPSS version 22 was used.

## Results

Between 1999 and 2008, 265,324 injuries were registered, and 3,368 persons were diagnosed with whiplash ICD 10 code S 13.4. Traffic accidents comprised 39 819 injuries and 2,809 of 3,368 patients in this study had a whiplash injury due to a traffic accidents. Average age for participating women was 34.0, median 32.0 with SD 16.0. Average age for men was 33.0 year, median 30.0 and SD 16.0. It turned out that women and men showed a difference in the mode of transport at the time of the traffic accident (p<0.001, Chi-square, Table 1).

**Table 1:** Mode of transport for patients with a whiplash injury caused by a traffic acciddent.

	All patients	Women	Men
Car	2405 (86%)	1300 (46%)	1105 (39%)
Heavy vehicle	124 (4.4%)	27 (0.96%)	97 (3.4%)
Other	135 (4.8%)	93 (3.3%)	42 (1.5%)
Motorbike	51 (1.8%)	2 (0.071%)	49 (1.7%)
Moped/bike/walk	94 (3.3%)	46 (1.6%)	48 (1.7%)
Summary	2809 (100%)	1468 (52%)	1341 (48%)

Four hundred fifty-seven patients with whiplash trauma (14%) sought care with a delay of at least three days (72 hours) and women sought care later than men (p = 0.00074, Table 2).

**Table 2:** Care-seeking behavior, treatment given and hospital stay after whiplash trauma (n = 3,368).

		Women (n = 1,719)	Men (n = 1,649)	P-value
**Sought care at primary health care**		884 (51%)	769 (47%)	0.0060*
**Patient delay between trauma and seeking care (days)** ^**b**^		3.2 (15)—0 (0–2)^§^	2.6 (11)—0 (0–1)	0.00074*
**Type of treatment**				0.69
	Discharged without treatment, [n (%)]^a^	107 (6.2%)	111 (6.8%)	
	Discharged after treatment, [n (%)]^a^	1.506 (88%)	1.423 (86%)	
	Admitted to hospital, [n (%)]^a^	106 (6,2%)	109 (6,4%)	
**Days admitted to hospital, [mean (SD)]**^**c**^		2.70 (1.9)	2.10 (1.7)	0.022*

*Significant at the p<0.05 level.

^**§**^ First figure mean values (standard deviation) second figure median (25^th^-75^th^ percentile).

^**a**^ Chi-square test.

^**b**^ Mann Whitney U-test.

^**c**^ Students T-test.

Forty-nine percent (1,653 of the 3368 with whiplash trauma) initially sought care at primary healthcare facility while 1,715 persons (51%) first sought care at a hospital. There was a statistically significant difference in care-seeking behavior between women and men (Table 2).

There was no difference between women and men in the type of treatment after trauma (Table 2). 215 patients (6.4%) were admitted to a hospital. Women were hospitalized for longer than men were (p = 0.022, Table 2). Twenty-four patients stayed at a hospital for more than three days. The 5 male patients admitted to a hospital for more than three days had other ICD 10 diagnoses: 2 commotio cerebri, 1 lumbar fracture and 1 ribcage contusion, 1 maxillary fracture, and 1 hand contusion and 1 radius fracture. Of the 19 female patients admitted for more than three days, 8 had other ICD 10 diagnoses: 2 commotio cerebri, 1 cervical fracture, 1 fracture of the upper arm, 1 contusion of the ribcage, 1 femoral contusion, 1 abrasion of the head, and 1 abrasion of the forearm. There were no significant differences between men and women in other comorbidities (Table 3).

**Table 3:** Diagnostic panorama in patients after a whiplash trauma.

	= = = = = = = = All patients = = = = = = =				= = = All patients Admitted To Hospital = = =			
Diagnosis	Women+Men	Women	Men	P- value	Women+Men	Women	Men	P- value
Only whiplash injury	2856 (85%)	1459 (43%)	1397 (41%)	0.90	94 (44%)	50 (23%)	44 (20%)	0.32
Whiplash injury & other injuries	512 (15%)	260 (7,8%)	252 (7,5%)	0.90	121(56%)	56 (26%)	65 (30%)	0.32
Whiplash injury total	3368 (100%)	1719 (51%)	1649 (49%)	------	215 (100%)	106 (49%)	109 (51%)	------
Contusion injury	387 (16%)	202 (6.0%)	185 (5.5%)	0.63	59 (27%)	29 (13%)	30 (14%)	0.98
Comutio Cerebri	90 (2,7%)	44 (1.3%)	46 (1.4%)	0.68	58 (27%)	26 (12%)	32 (15%)	0.42
Wounds injury	127 (3.8%)	56 (1.7%)	71 (2.1%)	0.11	18 (8.4%)	9 (4.2%)	9 (4.2%)	0.95

Female gender remained as an important predictor for patient delay even after adjusting for confounding variables (Table 4).

**Table 4:** Predictors for patients delay between trauma and seeking care (n = 3350).

	Spearman Rank Correlation				Multivariate linear regression^a^^,^^b^	
		number	r^a^	p-value^b^	β^c^	p-value
Age in decades		3350	0.083	<0.0001	**>0 (longer delay)**	**<0.0001**
Female gender (n = 1712)		3350	0.058	0.00074	**>0 (longer delay)**	**0.0037**
Work-related trauma (n = 1037)		3314	-0.036	0.039	<0 (shorter delay)	0.097
Traffic accident (n = 2395)		3350	-0.020	0.24		

^a^ The patients delay between trauma and seeking care (days) was transformed to ranked normal score of the dependent variable, using Blom’s formula.

^b^ Variables with p <0.05 in Spearman’s rank correlation were entered into the multivariate model.

^c^ The point estimate and confidence intervals for the beta coefficient as such makes no sense since the dependent variable is transformed as a rank. Hence, only it’s direction is indicated.

## Discussion

In this study of behavior after a whiplash trauma, we found that women sought healthcare later than men did, women sought care at primary care facilities slightly more often than men who more often went directly to a hospital. Nothing indicated that women had a milder injury than men did. Seeking care at the right level is important for optimal allocation of healthcare resources. During this ten-year period, only 6.4% of patients required hospital care. Therefore, it is surprising to see that 51% of patients initially sought care at a hospital, rather than visiting a primary healthcare facility.

### Gender and insurance claims

In this study, we can see a statistically significant gender difference where women seek healthcare later than men. This may also be clinically significant since it can potentially affect insurance outcomes, based on the theoretical link between care sought later and the willingness of insurance companies to award compensation. "The 72 hour rule” is commonly applied in connection with insurance claims in Sweden and Norway. Seeking care later (72 hours or more after trauma) is likely to reduce the probability of getting any compensation from insurance companies. As recently, as 2015 a study was commissioned by the insurance company Aviva to investigate what the UK can learn from overseas motor insurance companies when addressing rising claims for whiplash [32]. Frontier looked at five European countries, France, Germany, Norway, Spain and Sweden and suggested that the UK should also introduce the“72 hour rule” as a means of declining insurance claims [32]. *“Other countries like Norway and Sweden have limitation periods and also require medical reports to be obtained within short periods (72 hours)*. *MedCo could look to include this as part of the process*.*”* [32]. Information from our study opens the way for future economic studies investigating the gender implications of late care seeking and its influence on possible compensation in case of chronic WAD. This study showed gender differences with a clear disadvantage for women. Therefore, future guidelines addressing WAD needs to consider using information channels suitable to both women and men.

### Gender and choice of healthcare facility

This study found a statistically significant gender difference where women were more likely to initially seek care after a whiplash trauma at a primary healthcare facility compared to men. This gender difference is unlikely to be of any clinical significance for the single patient but might be of interest when planning allocation of health care resources.

One possible explanation for the difference in seeking level of care could be that in Swedish women visit a primary healthcare facility during pregnancy for antenatal care and postnatal care after childbirth, making it more likely that they would utilize a primary healthcare facility with other healthcare needs, like a whiplash injury [22]. Another explanation might be that women tend to be more burdened by guilt for having been involved in a traffic accident [33]. A further explanation could be women’s responsibility for the home and family means that women do not prioritize their own health, even in the presence of symptoms [34]. Finally, gender differences in health care seeking behavior may also be explained by that women and men are raised with different gender based expectations and structures within the family and labor market [35, 36].

The cultural gender norms mean men seek healthcare at hospital [37]. Similarly, the male role means that men are not ashamed of having been involved in a car accident [38]. According to Conell’s economical gender dimension, another alternative explanation for men attending healthcare early after trauma might be that men have the responsibility for insurance documents at home more often than women [38]. This might result in a greater understanding of the importance of early medical contact to gain adequate documentation and subsequent correct insurance compensation.

### Methodological strengths

The main strength in this study is that it is based on clinical encounters in the healthcare system and is not limited to data from an insurance company. Data quality was strengthened as all patients included were examined by the physician in charge who documented and registered a diagnosis according to ICD-10 after taking a history and doing a clinical examination. The quality of this database was closely monitored with regular checks of data quality between 1998 and 2008, and data from this period is of very high quality.

### Methodological weaknesses

One weakness is that there are no data regarding insurance claims or compensations in this study. Another weakness is that we have no information concerning how many women were mothers. We do know that the average age for women in this study was 34.0 years and the average age for first-time mothers in Sweden is 27–31 years. Hence, we may assume that a fair proportion of participating women also were mothers.

The missing trauma cases in the present study were 20%. There is no reason to believe that those who did not register were more seriously injured, although that is a possibility. Patients more seriously injured (Abbreviated Injury Scale, AIS 3–5) may have been registered as suffering from something other than neck distortion and whiplash, even if they also had those injuries. Awareness, knowledge and discussions of WAD in the society and among professionals has increased during the last 15 years making it less likely that symptoms indicative of a whiplash injury are ignored and not registered [10].

The level of WAD injury was not classified in the medical documentation, and it is not possible to do so retrospectively. Another limitation is that no follow-up was possible for individuals involved in a whiplash trauma. We can therefore not report the long-term fate or care of these patients. The study base, Skaraborg County, is not necessarily comparable with studies in cities with a population larger than 100,000 where traffic may be more intense.

## Conclusion

This study showed that gender differences exist regarding time to seek healthcare after whiplash trauma, even when considering other coexisting injuries or length of hospital stay. Although not directly investigated in this study, the present study suggests the 72-hour rule commonly used by insurance companies may create an unjustified gender inequality. Future studies should focus on investigating gender differences in success of insurance claims and gender differences in rehabilitation programs after traffic collisions.
